# Supplementary material for: Glucocorticoid receptor-PPARα axis in fetal mouse liver prepares neonates for milk lipid catabolism
Source: eLife. 2016 Jul 1;5:e11853. doi: 10.7554/eLife.11853 (PMC4963200; doi:10.7554/eLife.11853)

**Figure 2−source data 1.**

The dataset provides a list of PPARα-regulated genes (A) and pathways (B) in E19.5 and P2 mouse livers. Significant genes based on a false discovery rate < 0.05 were classified as regulated at both E19.5 and P2, in E19.5 only, or P2 only. The logarithmic fold change (logFC) cut-off value was set at 1.3. For each group, the significant enrichment of underlying KEGG, GO, and Reactome curated pathways was determined from the hypergeometric distribution and corrected for multiple comparisons.

*(A) PPARα-regulated genes in E19.5 and P2 mouse livers*


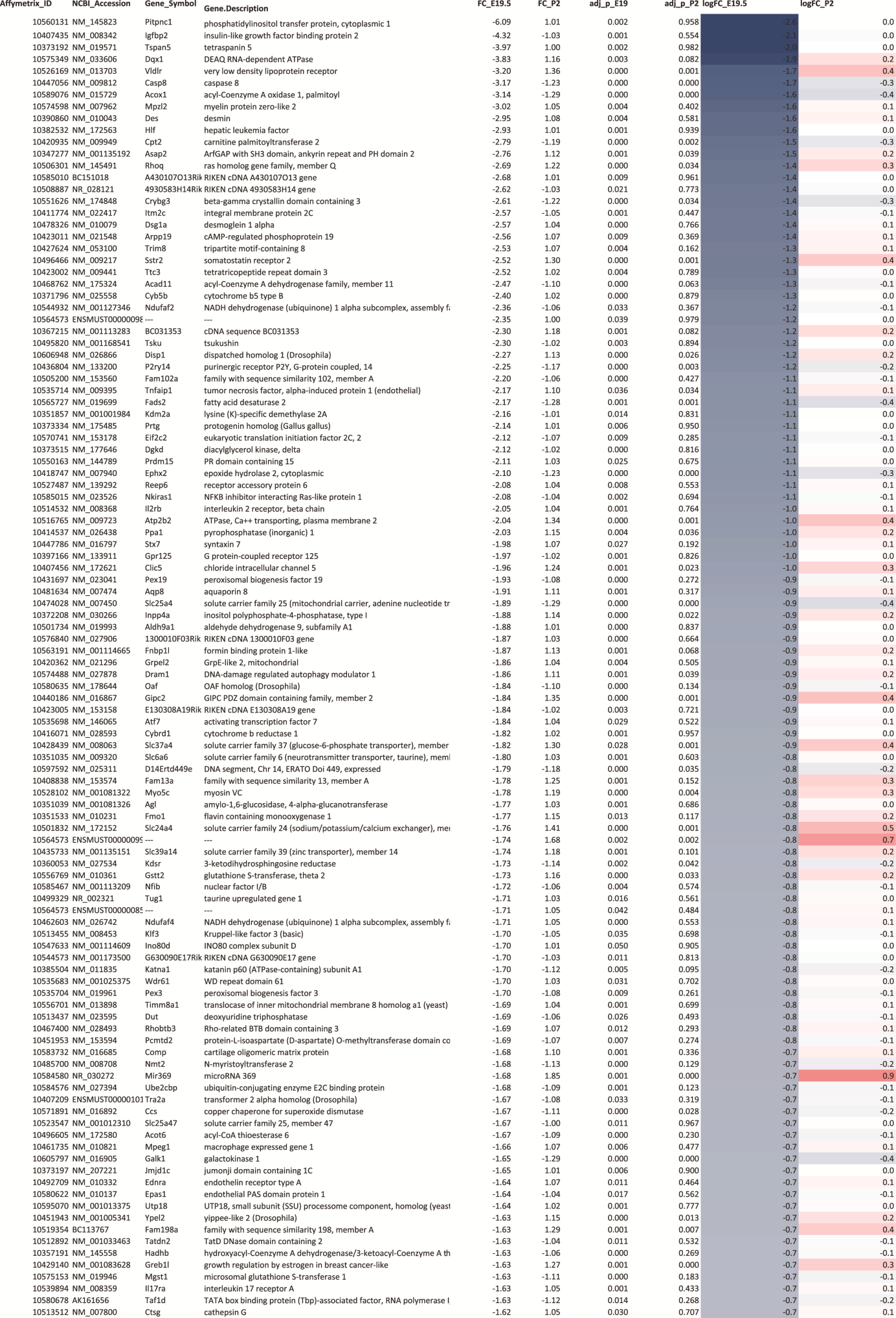


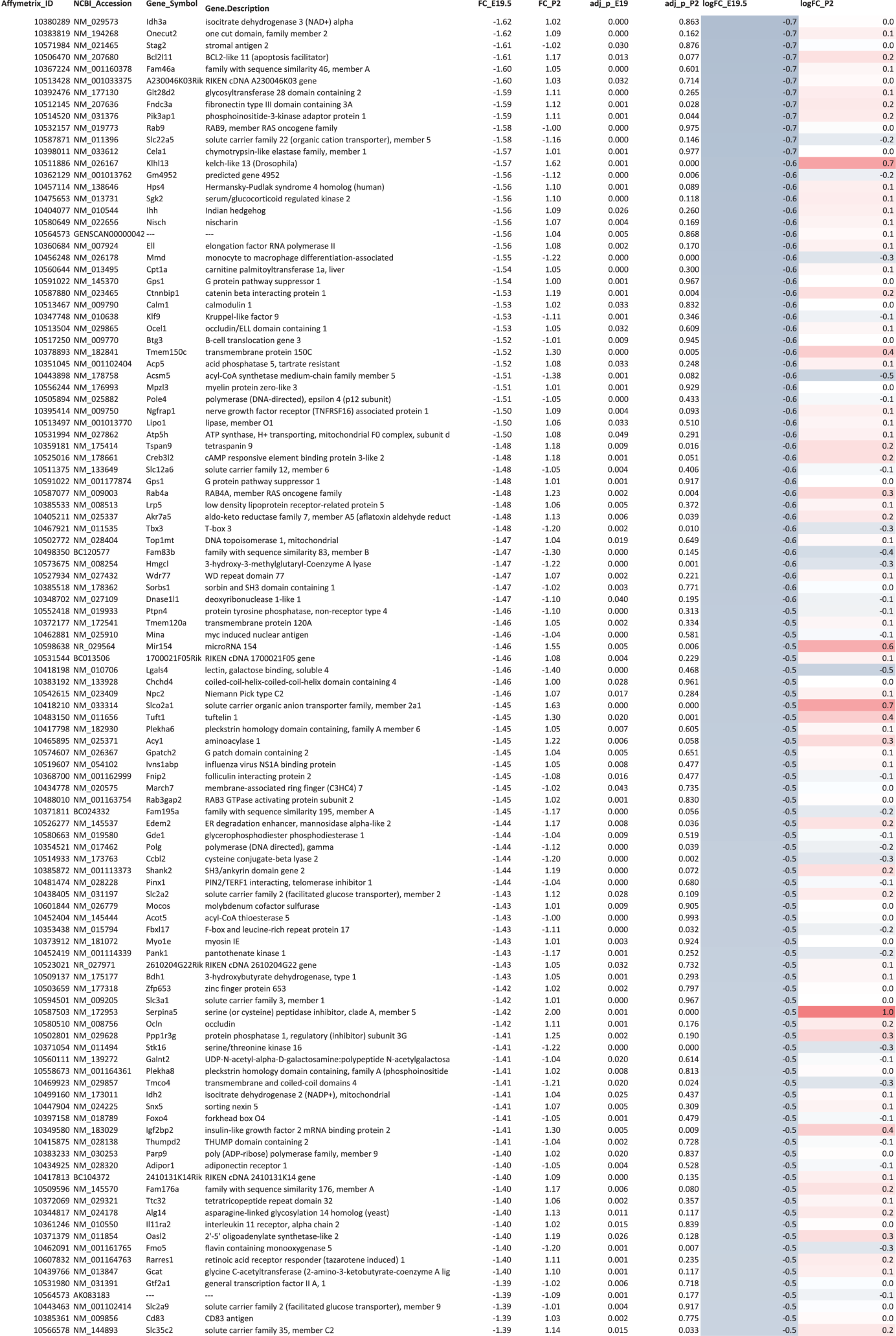


**
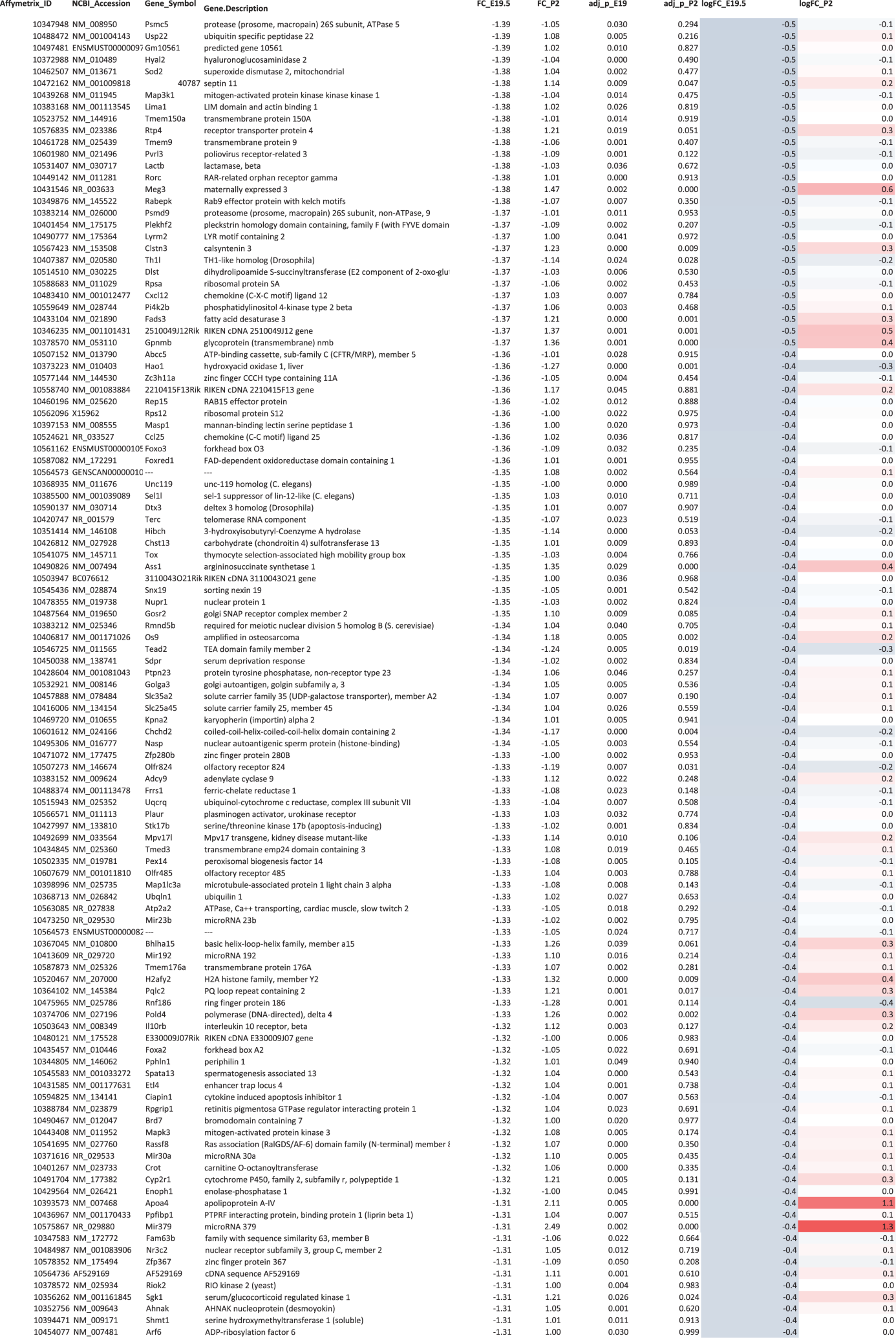
**

^
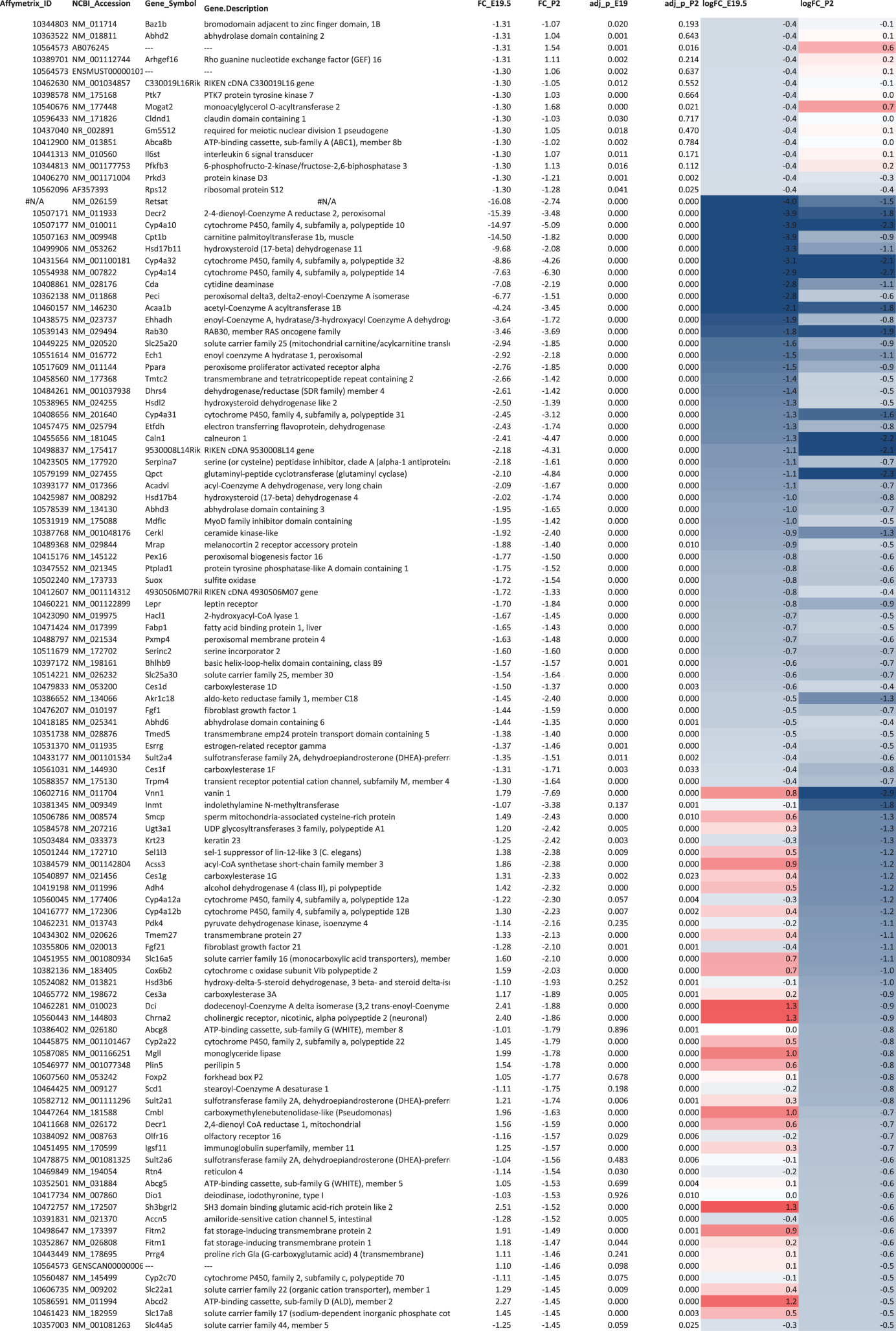
^


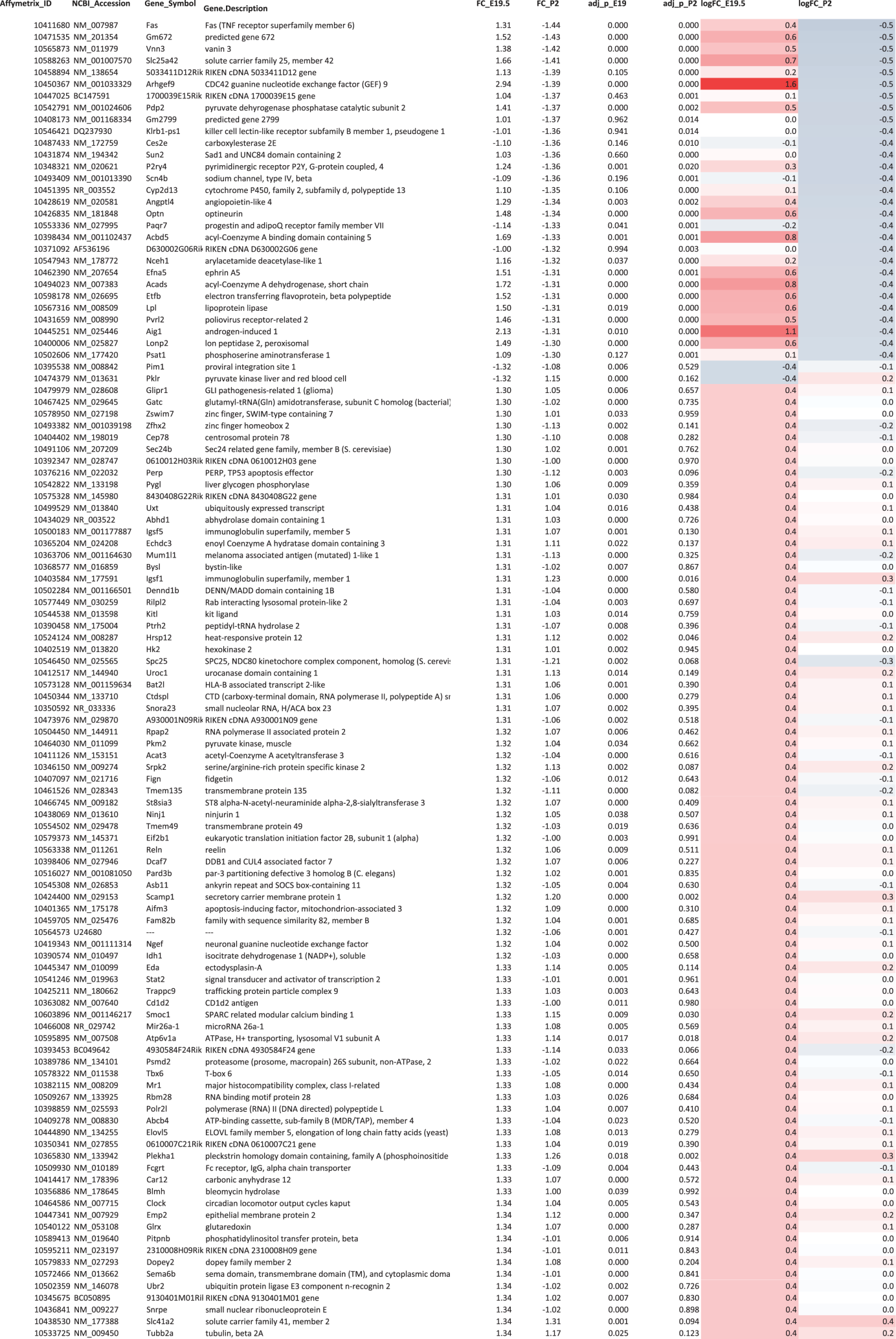


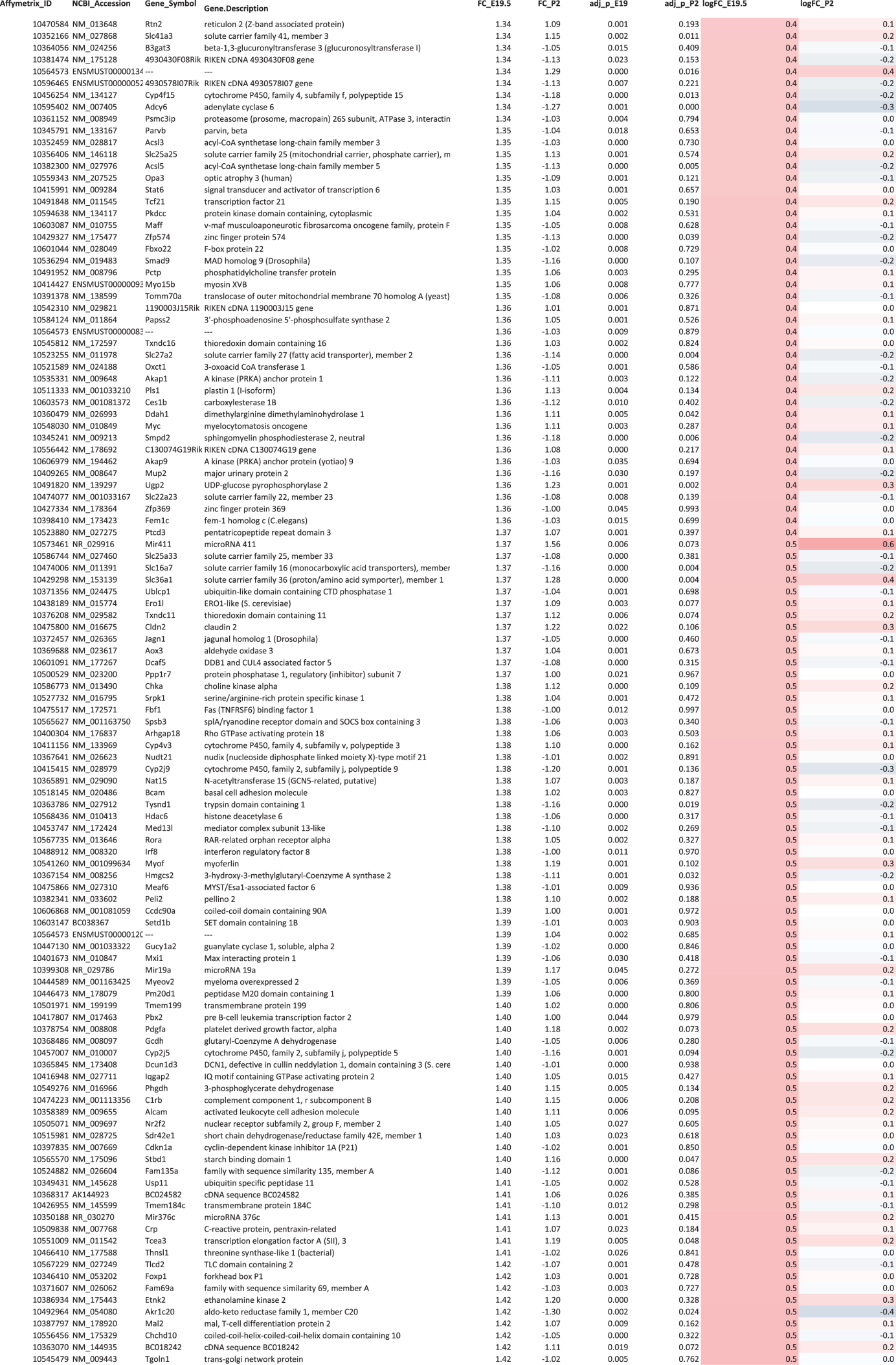

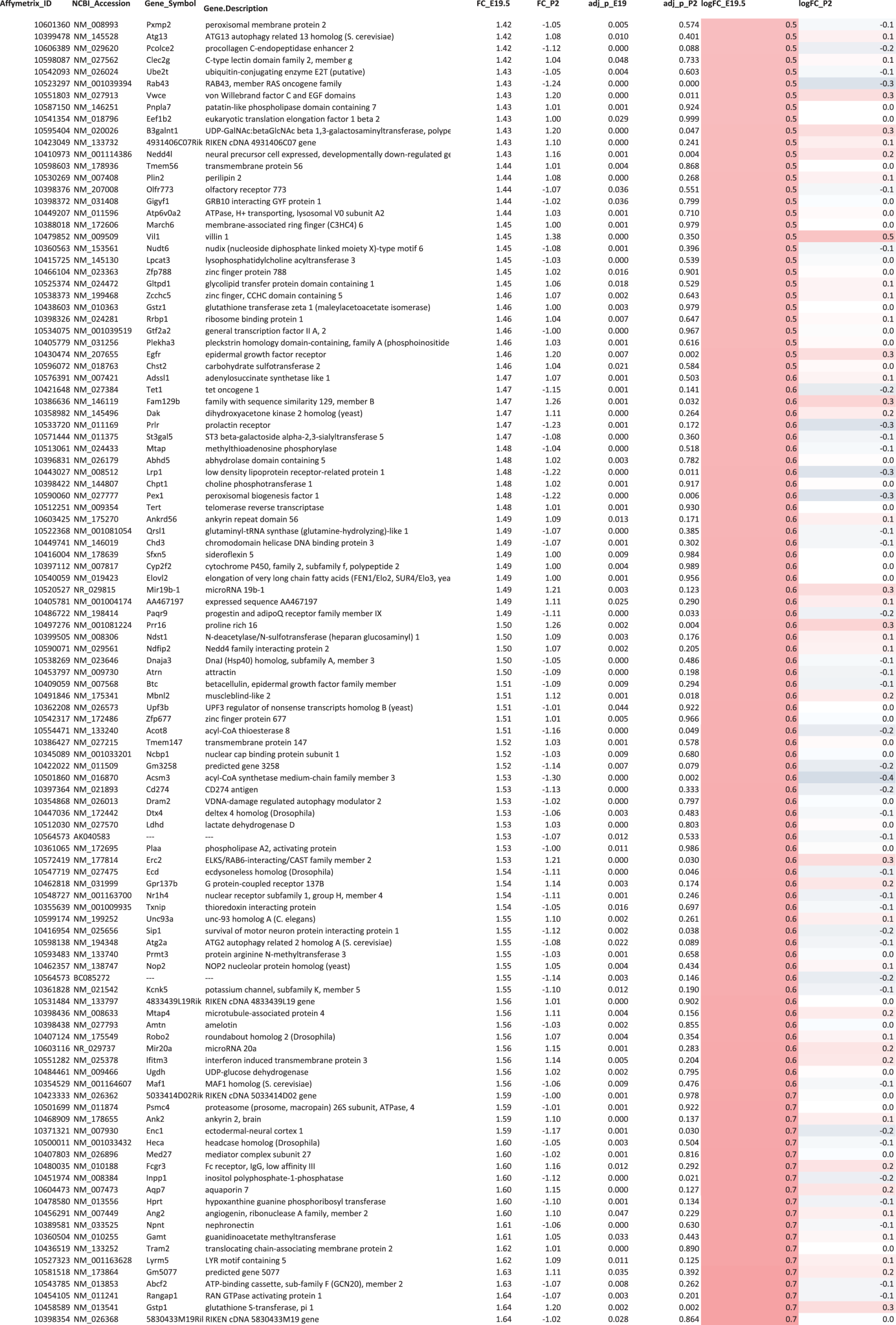

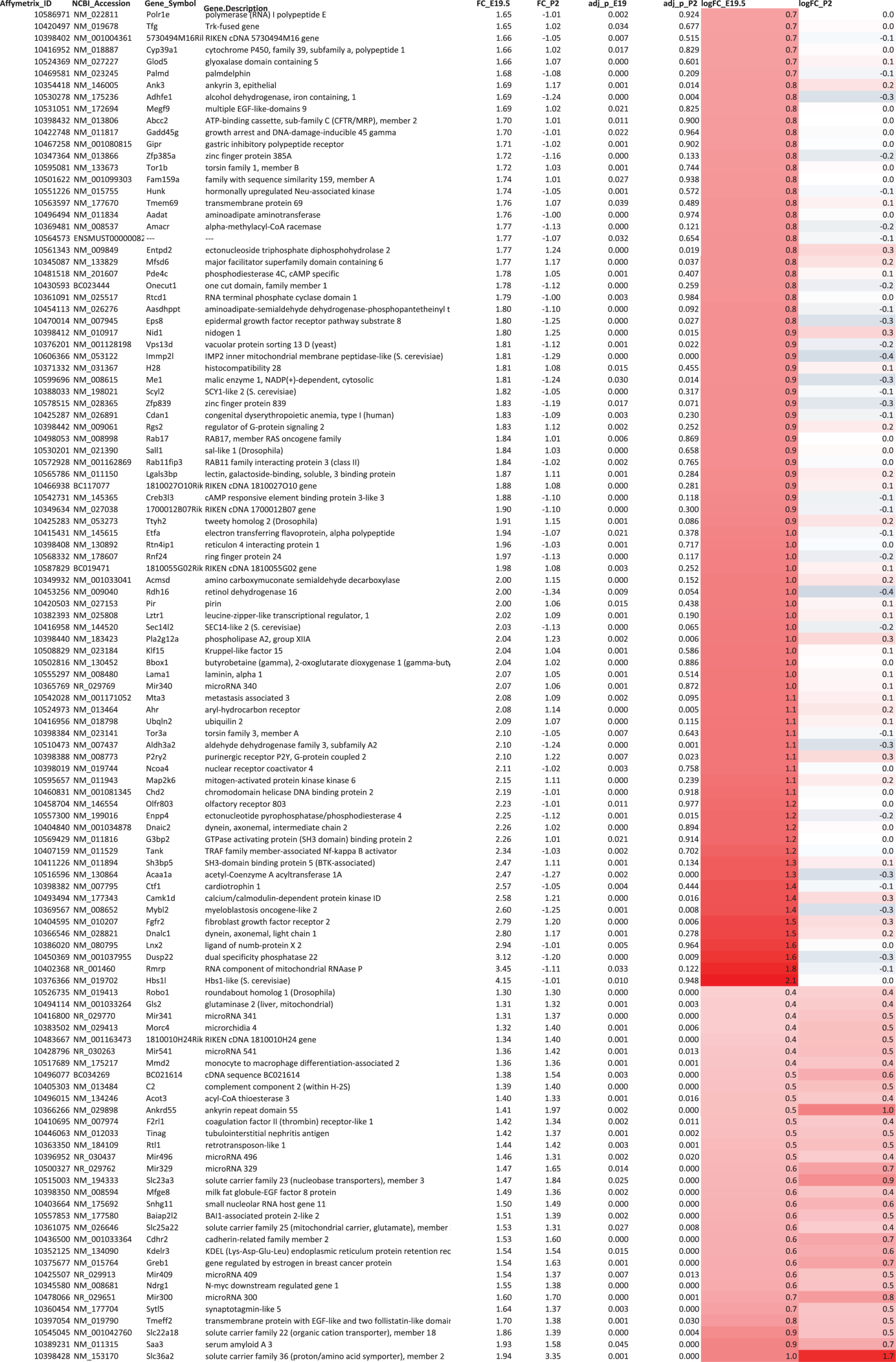

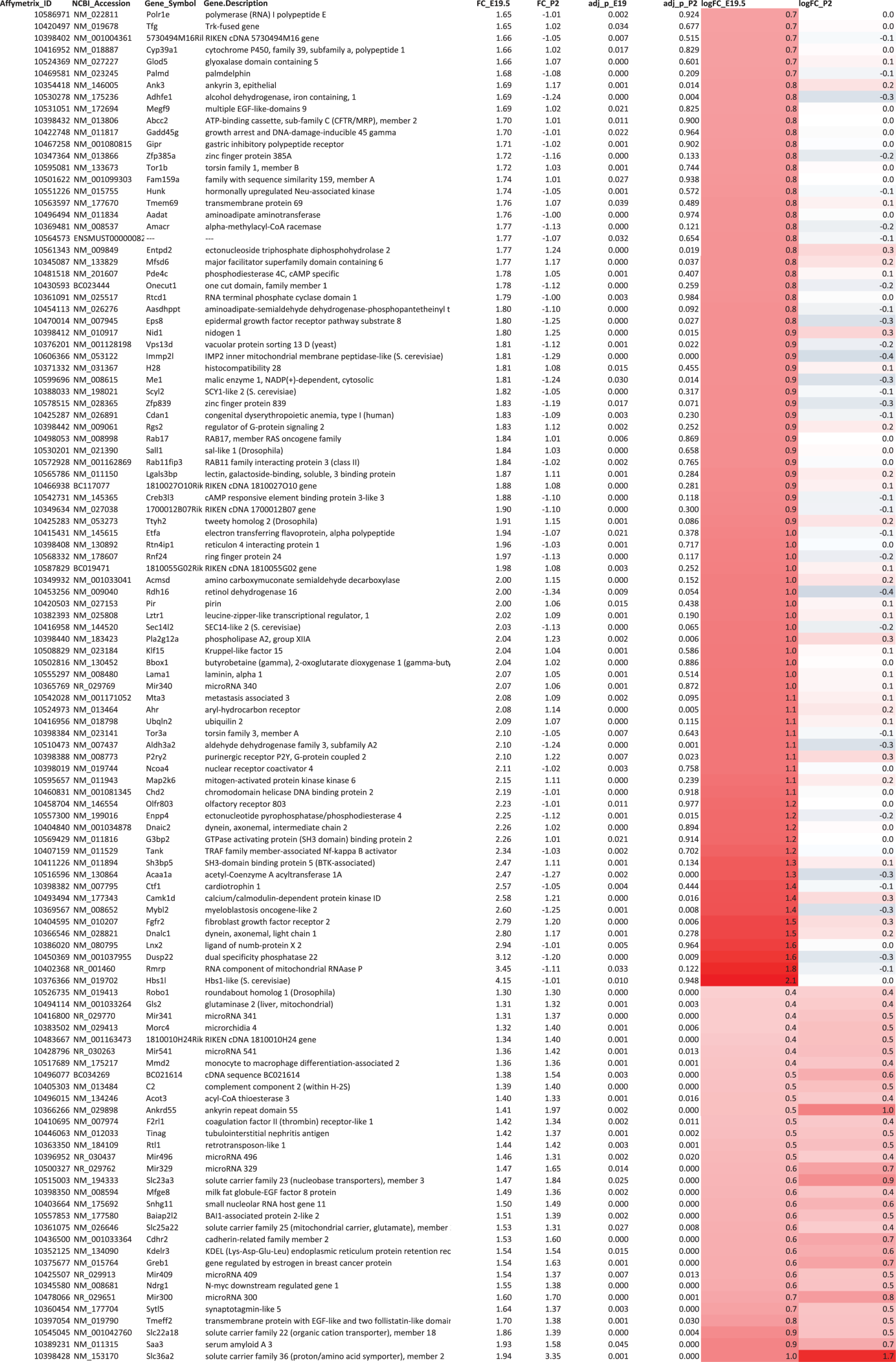

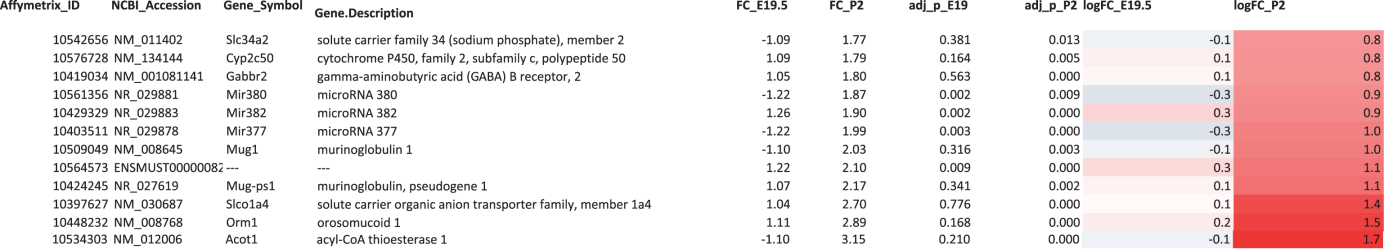


*(B) PPARα-regulated pathways in E19.5 and P2 mouse livers*


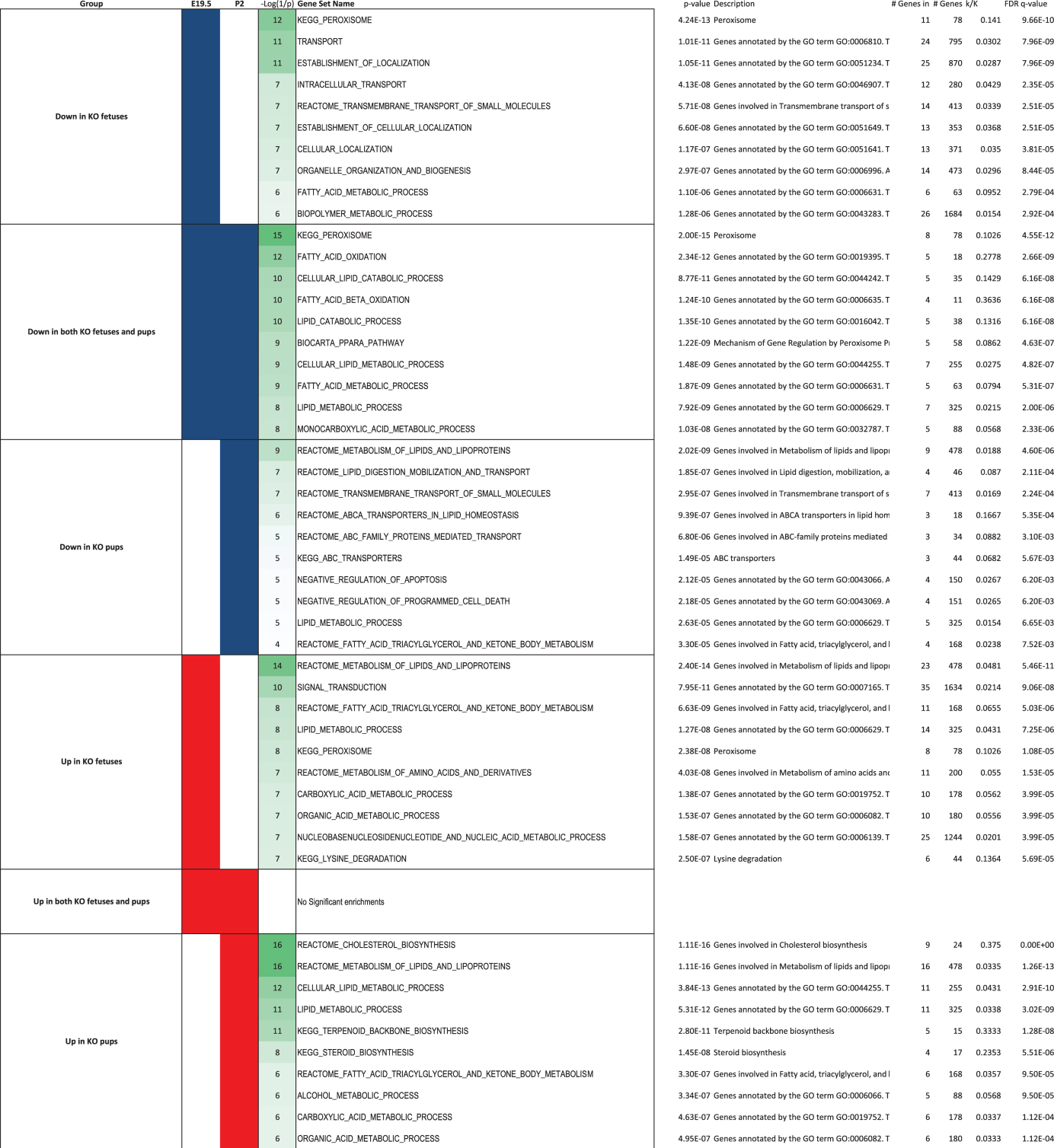

Supplement: Figure 2—source data 1. — The dataset provides a list of PPARα-regulated genes (A) and pathways (B) in E19.5 and P2 mouse livers. Significant genes based on a false discovery rate < 0.05 were classified as regulated at both E19.5 and P2, in E19.5 only, or P2 only. The logarithmic fold change (logFC) cut-off value was set at 1.3. For each group, the significant enrichment of underlying KEGG, GO, and Reactome curated pathways was determined from the hypergeometric distribution and corrected for multiple comparisons. DOI: http://dx.doi.org/10.7554/eLife.11853.005 [file elife-11853-fig2-data1.docx]
